# The genomic architecture of mastitis resistance in dairy sheep

**DOI:** 10.1186/s12864-017-3982-1

**Published:** 2017-08-16

**Authors:** G. Banos, G. Bramis, S. J. Bush, E. L. Clark, M. E.B. McCulloch, J. Smith, G. Schulze, G. Arsenos, D. A. Hume, A. Psifidi

**Affiliations:** 1 The Roslin Institute and Royal (Dick) School of Veterinary Studies, University of Edinburgh, Easter Bush, Midlothian, EH25 9RG UK; 2 Scotland’s Rural College, Edinburgh, Easter Bush, Midlothian, EH25 9RG UK; 3 School of Veterinary Medicine, Aristotle University of Thessaloniki, 54124 Thessaloniki, Greece; 4 School of Informatics, University of Bergen, 5008 Bergen, Norway; 5 Royal Veterinary College, University of London, AL9 7TA Hatfield, UK

**Keywords:** Genomic association, Sheep transcriptomic atlas, Milk transcriptome, Transcription factor binding sites, Mastitis, Somatic cell count, Total viable bacterial count, California mastitis test, Chios sheep, Dairy sheep

## Abstract

**Background:**

Mastitis is the most prevalent disease in dairy sheep with major economic, hygienic and welfare implications. The disease persists in all dairy sheep production systems despite the implementation of improved management practises. Selective breeding for enhanced mastitis resistance may provide the means to further control the disease. In the present study, we investigated the genetic architecture of four mastitis traits in dairy sheep. Individual animal records for clinical mastitis occurrence and three mastitis indicator traits (milk somatic cell count, total viable bacterial count in milk and the California mastitis test) were collected monthly throughout lactation for 609 ewes of the Greek Chios breed. All animals were genotyped with a custom-made 960-single nucleotide polymorphism (SNP) DNA array based on markers located in quantitative trait loci (QTL) regions for mastitis resistance previously detected in three other distinct dairy sheep populations.

**Results:**

Heritable variation and strong positive genetic correlations were estimated for clinical mastitis occurrence and the three mastitis indicator traits. SNP markers significantly associated with these mastitis traits were confirmed on chromosomes 2, 3, 5, 16 and 19. We identified pathways, molecular interaction networks and functional gene clusters for mastitis resistance. Candidate genes within the detected regions were identified based upon analysis of an ovine transcriptional atlas and transcriptome data derived from milk somatic cells. Relevant candidate genes implicated in innate immunity included *SOCS2, CTLA4, C6, C7, C9, PTGER4, DAB2, CARD6, OSMR, PLXNC1, IDH1, ICOS, FYB,* and *LYFR*.

**Conclusions:**

The results confirmed the presence of animal genetic variability in mastitis resistance and identified genomic regions associated with specific mastitis traits in the Chios sheep. The conserved genetic architecture of mastitis resistance between distinct dairy sheep breeds suggests that across-breed selection programmes would be feasible.

**Electronic supplementary material:**

The online version of this article (doi:10.1186/s12864-017-3982-1) contains supplementary material, which is available to authorized users.

## Background

Mastitis is an inflammation of the mammary gland usually caused by pathogens, mainly bacteria, developed in the gland cistern after penetration through the teat canal. It is the most prevalent and costly disease in the dairy industry due to reduced and discarded milk, early involuntary culling, veterinary service and labour costs [1, 2]. There is also the possibility for the spread of a zoonotic disease as well as the development of microbial resistance to antibiotics [1–3]. Mastitis compromises animal welfare causing pain, anxiety, restlessness and changes in feeding behaviour [4]. The issue of mastitis is a central element in the design and implementation of management schemes in dairy sheep farms. The notion is that for every case of confirmed clinical mastitis there are at least 40 other animals in the flock with subclinical mastitis. Reviews of management practices and practical approaches to control sheep mastitis have been published [5, 6].

The susceptibility of dairy animals to udder infection is heritable [2, 7–9] but the current knowledge of the complex physiological and cellular events that occur in the mammary gland in response to pathogens is limited. In cattle, the common mastitis pathogens*, Escherichia coli* (*E. coli*) and *Staphylococcus aureus* (*S. aureus*), activate the mammary immune system in different ways, influencing disease severity and chronicity [10]. The recruitment of leukocytes (neutrophils) in the udder in response to infection is measured as the milk somatic cell count (SCC) [11]. Breeding programmes based on animal pedigrees and SCC records (as a correlated trait for mastitis) have been implemented in dairy cattle worldwide [12] as well as the French Lacaune dairy sheep [13, 14]. Marker assisted selection or genomic selection based on genotyping of single nucleotide polymorphism (SNP) markers could enhance and expedite the efforts for genetic improvement.

Quantitative trait loci (QTLs) and SNPs associated with SCC have been previously identified based on linkage and linkage disequilibrium analyses of three distinct dairy sheep breeds (French Lacaune, Spanish Churra and Italian Sarda [15–19]). Several QTLs were common (i.e. located within 2 Mb regions) to two or more breeds in these studies. A custom-made 960-SNP DNA array for ovine mastitis resistance containing markers on six chromosomes (2, 3, 5, 12, 16 and 19) was then developed. The array was based on the common QTLs among breeds and some additional well-defined (i.e. confidence interval less than 0.5 Mb) QTL regions with large effects, as well as further genotyping with the Illumina OvineSNP800 Bead chip and re-sequencing of selected QTL regions (Additional file 1: Table S1) within an FP7 European funded project (http://cordis.europa.eu/result/rcn/163471_en.html). For the design of the custom-made array, SNPs were selected from both the 50 K and the 800 K SNP DNA arrays, as well as the available re-sequencing data; the array had an average density of 1 SNP every 23 Kb. The present study builds on results from previous studies on Lacaune, Churra and Sarda sheep [15–19] in order to dissect the genomic architecture of mastitis resistance in an independent sheep population, the Greek Chios breed. Genotyping was performed with the aforementioned mastitis-specific custom-made DNA array. We conducted variance component analyses to estimate genetic parameters and genomic association studies to identify genomic regions controlling mastitis. We also performed pathway analysis and examined gene expression and transcription factor binding site data to identify candidate genes within the relevant genomic regions.

## Results

### Principal component analysis

French Lacaune, Spanish Churra, Italian Sarda and Greek Chios were among the sheep breeds analysed within the framework of the sheep HapMap project [20]. Principal component analysis (PCA) was performed to examine the relatedness among these dairy breeds using genotypes obtained with the Illumina OvineSNP50 Beadchip within the HapMap project. According to the results, the four dairy breeds are genetically distinct with the first and second PCA clusters explaining 14.4% and 9.9% of the variance, respectively (Additional file 2: Figure S1). Chios is equally distant from any one of the other three breeds, with an average pairwise genetic distance (*D*_st_) of 0.33, confirming its independent population status for the purposes of the present study.

### Descriptive statistics of phenotypes

**Fig. 1:**
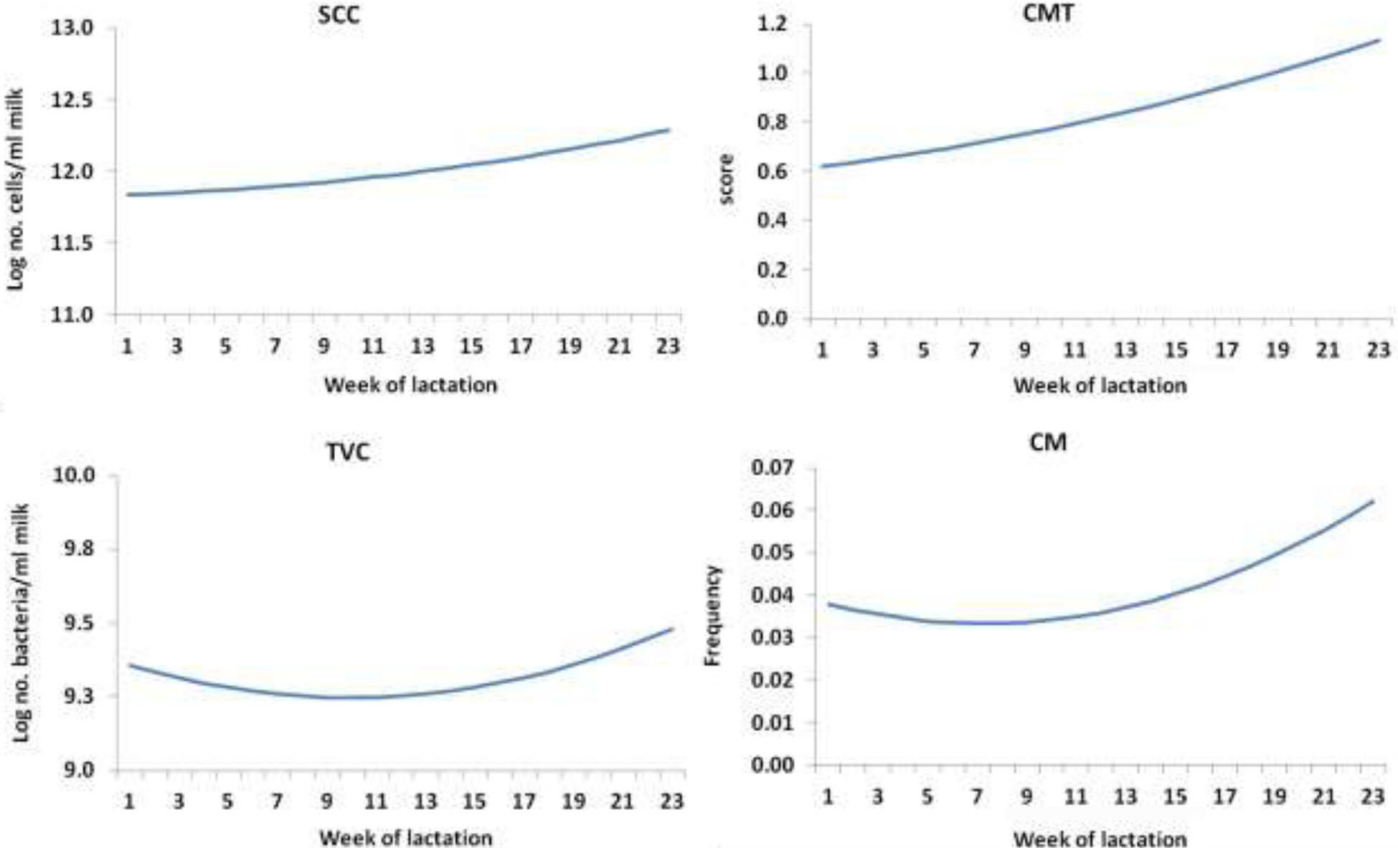
Smoothed fixed curves of the mastitis traits studied in Chios sheep by week of lactation. Curves for milk somatic cell count (SCC), California mastitis test (CMT), total viable bacterial count in milk (TVC) and clinical mastitis occurrence (CM) are presented

Descriptive statistics for SCC, California mastitis test (CMT) and total viable bacterial count in milk (TVC) are listed in Additional file 3: Table S2. The average frequency of clinical mastitis occurrence (CM) was 5.1% in our studied population. Gram positive (58% *Coagulase negative Staphylococci*, 22% *S. aureus* and 7.7% *Streptococcus* spp) bacteria were mainly responsible for CM. Nevertheless, Gram negative bacteria (9.3% *Pasteurella* spp. and 3% *Proteus*) were also isolated from the milk samples. Smoothed curves illustrating progression for each trait throughout lactation, after adjusting for all relevant sources of systematic variation, are shown in Fig. 1. These curves were based on second order polynomial functions of time (week of lactation) fitted in mixed model analyses of the studied traits. Higher order polynomials did not improve the model fit as attested to by the Wald statistic. As expected, values of SCC and CMT generally increased as lactation progressed [21]. TVC and CM values first showed a relative decrease until week 11 of lactation and then started gradually increasing.

### Genetic parameters

Estimates of heritability and repeatability of the studied traits (Table 1) based on monthly records were derived for the entire lactation as well as different stages of lactation defined as early (weeks of lactation 1–7), mid (weeks 8–17) and late (weeks 18–23). Because of the binary nature of CM, only overall lactation heritability and repeatability estimates were possible to derive for this trait. Low to moderate heritabilities (0.09–0.18) were estimated for all the mastitis traits; higher heritability estimates were observed in all cases toward the end of lactation. Moreover, the genetic correlations between the different lactation stages were positive for all studied traits (Additional file 4: Table S3). However, the genetic correlations between early and late lactation were low to moderate (0.26–0.56) suggesting that somewhat distinct genetic mechanisms may control mastitis resistance at different lactation stages. The three mastitis indicator traits (SCC, TVC and CMT) were correlated, both phenotypically and genetically, with each other and with CM, suggesting that any of the three indirect mastitis traits would be a useful predictor of CM. The genetic and phenotypic correlation estimates between traits are presented in Table 2.

**Table 1:** Heritability (h^2^) and repeatability (r) estimates for four mastitis traits studied in Chios sheep, by stage of lactation

Trait	Parameter	Early lactation (weeks 1–7)	Mid lactation (weeks 8–17)	Late lactation (weeks 18–23)	Overall lactation (weeks 1–23)
SCC	h^2^	0.10 (0.05)	0.09 (0.05)	0.18 (0.05)	0.11 (0.05)
	r	0.55 (0.03)	0.54 (0.02)	0.66 (0.02)	0.58 (0.02)
CMT	h^2^	0.12 (0.06)	0.10 (0.05)	0.17 (0.06)	0.12 (0.06)
	r	0.60 (0.03)	0.59 (0.02)	0.68 (0.02)	0.62 (0.02)
TVC	h^2^	0.09 (0.05)	0.08 (0.04)	0.15 (0.05)	0.09 (0.05)
	r	0.28 (0.04)	0.27 (0.03)	0.34 (0.04)	0.33 (0.04)
CM	h^2^	NA	NA	NA	0.18 (0.10)
	r	NA	NA	NA	0.35 (0.06)

*SCC* log-transformed milk somatic cell count; *CMT* California mastitis test; *TVC* log-transformed total viable bacterial count in milk; *CM* clinical mastitis occurrence (0/1, analysed with a logit function); *NA* non-applicable; standard errors in parentheses

**Table 2:** Estimates of genetic (above diagonal) and phenotypic (below diagonal) correlations between the mastitis traits studied in Chios sheep (overall lactation)

	SCC	CMT	TVC	CM
SCC		0.77 (0.11)	0.56 (0.12)	0.35 (0.16)
CMT	0.85 (0.02)		0.21 (0.12)	0.36 (0.17)
TVC	0.64 (0.02)	0.65 (0.02)		0.35 (0.17)
CM	0.35 (0.03)	0.39 (0.03)	0.33 (0.03)	

*SCC* log-transformed milk somatic cell counts; *CMT* California mastitis test; *TVC* log-transformed total viable bacterial count in milk; *CM* clinical mastitis occurrence (0/1); standard errors in parentheses

### Genomic association studies

**Fig. 2:**
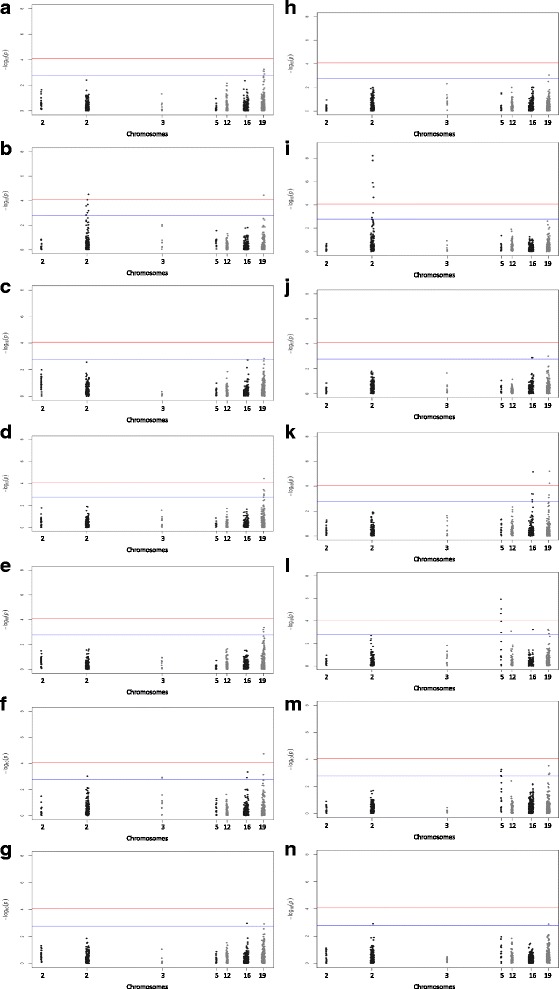
Manhattan plots displaying the genomic association results of the mastitis traits studied in Chios sheep. Manhattan plots for milk somatic cell count (SCC) in early (**a**), late (**b**), and overall (**c**) lactation; for California mastitis test (CMT) in early (**d**), mid (**e**), late (**f**), and overall (**g**) lactation; for total viable bacterial count in milk (TVC) in early (**h**), late (**i**), and overall (**j**) lactation; for clinical mastitis occurrence (CM) in early (**k**), mid (**l**), late (**m**), and overall (**n**) lactation. Chromosome location is plotted against -log_10_(P). *Red* and *blue lines*, respectively, are thresholds for significance post-Bonferroni correction (*P* < 0.05) and for suggestive significance (accounting for one false positive per genome scan). No significant results were identified for TVC and SCC in mid lactation

Genomic association studies were conducted separately for early, mid, late and overall lactation for each trait. Multidimensional scaling analysis (MDS) revealed no substructure in the Chios population. In general, similar genomic associations were detected for CM and the three mastitis indicator traits but distinct associations were observed in the different lactation stages. SNPs significantly associated with the studied mastitis traits are shown in Table 3. Five (four at genome-wide and one at suggestive genome-wide significant level) of the seven QTLs previously identified in other dairy breeds were verified in the independent Chios population (Additional file 1: Table S1). Q-Q plots supported the genomic analyses results. Manhattan plots displaying these association results are shown in Fig. 2; the corresponding Q-Q plots are shown in Additional file 5: Figure S2.

**Table 3:** List of SNPs associated with clinical mastitis occurrence and three mastitis indicator traits in Chios sheep

Trait	Lactation stage	SNP	^a^Chr(position)	GAS	a	d	PV_A_	PV_P_	p	q
				*P*-value	(*P*-value)	(*P*-value)				
SCC	early (weeks 1–7)	oar3_OAR19_27,830,713	19(27830713)	5.54E-04	−0.39(0.00)	−0.09(0.42)	0.36	0.04	0.62	0.38
		oar3_OAR19_27,397,476	19(27397476)	7.74E-04	−0.41(0.03)	0.32(0.15)	0.38	0.04	0.34	0.66
		OAR19_29193780	19(27558690)	8.00E-04	0.46(0.00)	0.30(0.04)	0.4	0.04	0.75	0.25
		OAR19_29776311	19(28134366)	1.28E-03	0.33(0.09)	0.09(0.26)	0.12	0.01	0.87	0.13
	late (weeks 18–23)	**oar3_OAR2_208,650,955**	**2(208650955)**	**2.31E-05**	**−0.24(0.07)**	**−0.52(0.00)**	**0.19**	**0.01**	**0.71**	**0.29**
		oar3_OAR2_206,805,418	2(206805418)	8.25E-05	−0.27(0.00)	0.45(0.13)	0.07	0	0.99	0.01
		OAR2_219641896	2(207420807)	1.65E-04	−0.14(0.46)	0.22(0.25)	0.01	0	0.83	0.17
		OAR2_216496832	2(204569747)	1.89E-04	−0.22(0.04)	0.01(0.91)	0.11	0.01	0.38	0.62
		oar3_OAR2_208,711,940	2(208711940)	6.73E-04	0.11(0.41)	−0.68(0.00)	0.18	0	0.19	0.81
		oar3_OAR2_207,141,059	2(207141059)	9.52E-04	−0.14(0.00)	−0.84(0.00)	0.14	0	0.96	0.04
		oar3_OAR2_205,201,880	2(205201880)	1.44E-03	−0.28(0.01)	0.03(0.79)	0.08	0.01	0.64	0.36
		oar3_OAR2_204,683,757	2(204683757)	1.51E-03	−0.20(0.04)	0.09(0.42)	0.04	0.01	0.49	0.51
		**oar3_OAR19_27,100,147**	**19(27100147)**	**2.75E-05**	**0.52(0.02)**	**0.63(0.00)**	**0.39**	**0.03**	**0.85**	**0.15**
	overall (weeks 1–23)	OAR16_38842323	16(35633299)	1.69E-03	−0.18(0.01)	0.12(0.13)	0.04	0	0.8	0.2
		oar3_OAR19_27,830,713	19(27830713)	1.45E-03	−0.21(0.01)	0.15(0.16)	0.09	0.01	0.62	0.38
CMT	early (weeks 1–7)	oar3_OAR19_27,397,476	19(27397476)	3.67E-04	−0.36(0.00)	0.07(0.50)	0.28	0.03	0.34	0.66
		oar3_OAR19_27,100,147	19(27100147)	3.93E-04	0.57(0.06)	0.33(0.31)	0.39	0.05	0.85	0.15
		OAR19_29776311	19(28134366)	4.78E-04	0.37(0.05)	0.10(0.60)	0.15	0.02	0.87	0.13
		OAR19_29193780	19(27558690)	9.22E-04	0.42(0.00)	0.21(0.12)	0.31	0.04	0.75	0.25
		oar3_OAR19_27,723,161	19(27723161)	1.18E-03	0.34(0.04)	0.11(0.54)	0.14	0.02	0.85	0.15
	mid (weeks 8–17)	oar3_OAR19_27,830,713	19(27830713)	4.31E-04	−0.20(0.01)	0.09(0.35)	0.11	0.01	0.62	0.38
		OAR19_27896777	19(26442150)	6.20E-04	0.61(0.01)	0.15(0.56)	0.19	0.04	0.91	0.09
		oar3_OAR19_27,264,253	19(27264253)	9.35E-04	0.20(0.01)	−0.02(0.79)	0.11	0.01	0.59	0.41
	late (weeks 18–23)	oar3_OAR2_206,805,418	2(206805418)	9.71E-04	−0.19(0.00)	0.29(0.28)	0.03	0	0.99	0.01
		oar3_OAR3_130,101,204	3(130101204)	1.18E-03	0.12(0.00)	0.14(0.15)	0.01	0	0.9	0.1
		OAR3_130,096,528.3SR	3(130096528)	1.32E-03	0.93(0.17)	0.98(0.16)	0.43	0.07	0.9	0.1
		oar3_OAR16_35,319,166	16(35319166)	4.60E-04	−0.22(0.12)	0.26(0.16)	0.04	0.01	0.81	0.19
		OAR16_37125504	16(34187287)	1.21E-03	−0.69(0.02)	−0.38(0.23)	0.24	0.04	0.89	0.11
		**oar3_OAR19_27,100,147**	**19(27100147)**	**1.79E-05**	**0.63(0.00)**	**0.79(0.00)**	**0.27**	**0.05**	**0.85**	**0.15**
		oar3_OAR19_26,711,220	19(26711220)	7.13E-04	0.08(0.00)	−0.03(0.74)	0.01	0	0.93	0.07
		oar3_OAR19_27,830,713	19(27830713)	1.16E-03	−0.20(0.01)	0.09(0.35)	0.05	0.01	0.62	0.38
		OAR16_37125504	16(34187287)	1.21E-03	−0.69(0.02)	−0.38(0.23)	0.24	0.04	0.89	0.11
	overall (weeks 1–23)	OAR16_37125504	16(34187287)	1.08E-03	−0.22(0.26)	0.12(0.56)	0.04	0.01	0.89	0.11
		oar3_OAR19_27,100,147	19(27100147)	1.09E-03	0.36(0.03)	0.42(0.01)	0.51	0.02	0.85	0.15
		oar3_OAR19_27,830,713	19(27830713)	1.16E-03	−0.18(0.00)	0.09(0.21)	0.07	0.01	0.62	0.38
		oar3_OAR19_27,830,713	19(27830713)	8.81E-04	−0.27(0.00)	0.08(0.37)	0.2	0.04	0.62	0.38
TVC	early (weeks 1–7)	**OAR2_219801849**	**2(207575434)**	**5.81E-09**	**0.27(0.00)**	**−0.23(0.03)**	**0.33**	**0.02**	**0.25**	**0.75**
	late (weeks 18–23)	**OAR2_219838408_X**	**2(207609417)**	**1.49E-08**	**0.22(0.03)**	**−0.17(0.11)**	**0.22**	**0.02**	**0.26**	**0.74**
		**OAR2_219641896**	**2(207420807)**	**1.26E-06**	**−0.15(0.27)**	**−0.08(0.59)**	**0.07**	**0.01**	**0.83**	**0.17**
		**oar3_OAR2_209,240,636**	**2(209240636)**	**2.85E-06**	**−0.19(0.51)**	**−0.32(0.09)**	**0.05**	**0**	**0.93**	**0.07**
		**oar3_OAR2_208,650,955**	**2(208650955)**	**2.26E-05**	**−0.12(0.18)**	**−0.32(0.00)**	**0.07**	**0**	**0.71**	**0.29**
		oar3_OAR2_206,805,418	2(206805418)	4.11E-04	−0.15(0.00)	0.28(0.19)	0.03	0	0.99	0.01
		OAR2_220844191	2(208597501)	4.81E-04	−0.10(0.22)	−0.16(0.10)	0.05	0	0.69	0.31
		oar3_OAR2_206,036,682	2(206036682)	1.24E-03	−0.49(0.00)	−0.50(0.00)	0.12	0.01	0.84	0.16
	overall (weeks 1–23)	OAR16_38842323	16(35633299)	1.27E-03	0.05(0.00)	−0.01(0.84)	0.06	0	0.8	0.2
		OAR16_37125504	16(34187287)	1.32E-03	−0.30(0.01)	−0.13(0.29)	0.15	0.01	0.89	0.11
		OAR19_27896777	19(26442150)	9.83E-04	0.16(0.16)	−0.03(0.79)	0.07	0	0.91	0.09
CM		**OAR16_39143709**	**16(35930429)**	**6.98E-06**	**−1.72(0.36)**	**0.41(0.52)**	**0.25**	**0.05**	**0.54**	**0.46**
	early (weeks 1–7)	OAR16_37226202	16(34279856)	4.16E-04	2.04(0.02)	−0.80(0.39)	0.28	0.05	0.51	0.49
		s74559	16(35801129)	4.41E-04	3.50(0.53)	2.81(0.61)	0.33	0.02	0.58	0.42
		OAR16_37419505	16(34392335)	1.21E-03	−3.27(0.06)	1.74(0.16)	0.19	0.05	0.62	0.38
		OAR16_37539041	16(34550197)	1.76E-03	1.60(0.01)	−1.17(0.13)	0.26	0.05	0.39	0.61
		**oar3_OAR19_28,110,151**	**19(28110151)**	**6.23E-06**	**0.98(0.04)**	**0.74(0.12)**	**0.25**	**0.05**	**0.86**	**0.14**
		**OAR19_29776311**	**19(28134366)**	**5.92E-05**	**1.02(0.05)**	**0.88(0.13)**	**0.25**	**0.04**	**0.87**	**0.13**
		OAR19_29193780	19(27558690)	4.95E-04	3.02(0.76)	2.66(0.79)	0.21	0.04	0.75	0.25
		oar3_OAR19_27,018,360	19(27018360)	8.26E-04	−1.11(0.05)	0.95(0.12)	0.26	0.05	0.46	0.54
		**OAR5_104431404**	**5(95881956)**	**1.18E-06**	**0.82(0.00)**	**0.29(0.39)**	**0.35**	**0.06**	**0.6**	**0.4**
	mid (weeks 8–17)	**oar3_OAR5_96,114,753**	**5(96114753)**	**8.66E-06**	**0.87(0.00)**	**0.41(0.25)**	**0.39**	**0.07**	**0.59**	**0.41**
		**OAR5_104389335_X**	**5(95841910)**	**2.28E-05**	**1.02(0.02)**	**−1.01(0.06)**	**0.27**	**0.09**	**0.64**	**0.36**
		oar3_OAR5_96,077,046	5(96077046)	1.08E-04	0.59(0.03)	0.89(0.05)	0.12	0.03	0.41	0.59
		oar3_OAR5_96,062,963	5(96062963)	1.07E-03	0.44(0.07)	−0.22(0.14)	0.12	0.02	0.43	0.57
		OAR16_37226202	16(34279856)	1.26E-03	2.02(0.02)	1.27(0.03)	0.21	0.04	0.51	0.49
		OAR16_37539041	16(34550197)	1.94E-03	1.60(0.01)	−1.17(0.13)	0.26	0.05	0.39	0.61
		oar3_OAR19_26,565,985	19(26565985)	5.65E-04	−0.70(0.04)	0.32(0.48)	0.25	0.05	0.52	0.48
		oar3_OAR19_26808003	19(268,080,030	6.95E-04	−0.58(0.05)	−0.07(0.97)	0.14	0.02	0.74	0.26
		OAR19_29482474	19(27864985)	1.30E-03	−3.49(0.86)	−3.33(0.87)	0.23	0.04	0.1	0.9
	late (weeks 18–23)	oar3_OAR5_96,114,753	5(96114753)	1.81E-03	1.00(0.03)	0.81(0.10)	0.42	0.08	0.59	0.41
		OAR5_104431404	5(95881956)	2.89E-04	0.98(0.03)	0.71(0.15)	0.4	0.07	0.6	0.4
		OAR5_104389335_X	5(95841910)	9.99E-04	0.47(0.04)	0.66(0.17)	0.21	0.02	0.64	0.36
		oar3_OAR5_96,114,753	5(96114753)	1.14E-03	0.62(0.01)	0.32(0.25)	0.24	0.04	0.59	0.41
		OAR19_29776311	19(28134366)	1.08E-03	1.08(0.00)	1.07(0.01)	0.28	0.05	0.87	0.13
		oar3_OAR19_27,264,253	19(27264253)	1.31E-03	1.00(0.03)	0.23(0.64)	0.42	0.08	0.59	0.41
		oar3_OAR19_28,110,151	19(28110151)	1.53E-04	0.98(0.05)	0.74(0.21)	0.25	0.05	0.86	0.14
		oar3_OAR19_26,565,985	19(26565985)	5.21E-04	−0.42(0.06)	−0.06(0.82)	0.1	0.02	0.52	0.48
		OAR19_29193780	19(27558690)	1.35E-03	0.56(0.10)	0.08(0.82)	0.15	0.02	0.75	0.25
	overall (weeks 1–23)	oar3_OAR2_207,141,059	2(207141059)	1.25E-03	−0.24(0.00)	−1.95(0.01)	0.1	0	0.96	0.04
		oar3_OAR19_27,264,253	19(2726253)	1.35E-03	0.88(0.00)	0.11(0.71)	0.36	0.07	0.59	0.41

*SCC* log-transformed milk somatic cell counts; *CMT* California mastitis Test; *TVC* total viable bacterial count in milk; *CM* occurrence of clinical mastitis

^a^Chr(position) is based on the Oar v3.1 assembly; GAS P–value: *P*-value from genomic association study (genome-wide significant in bold, suggestive significance otherwise); a: additive allele substitution effect; d: dominance effect; PV_A_: proportion of the genetic variance explained by the SNP; PV_P_: proportion of the phenotypic variance explained by the SNP; p and q allelic frequencies. No significant associations were identified for TVC and SCC in mid lactation

All significant SNP markers that were identified in the genomic association study had also a significant effect on the corresponding mastitis traits in the ensuing mixed model analyses based on the pedigree genetic relationship matrix among animals. The additive and dominance genetic effects, and the proportion of the total genetic and phenotypic variance explained by each of these SNPs, are summarised in Table 3. Individual significant SNP markers explained up to 27%, 39%, 33% and 39% of the genetic variance of CMT, SCC, TVC and CM, respectively. The significant markers corresponding to the same QTL were in linkage disequilibrium (LD as *r* ^*2*^ = 0.20–0.97), implying that they likely correspond to the same causative variation (Additional file 6: Table S4). Only small LD blocks (less than 200 kb length) were visualised with Haploview, probably due to a high number of recombination events having taken place in the outbred Chios population (Additional file 7: Figure S3). The significant markers were located in close proximity to each other, with most of them being inside neighbouring small LD blocks, and spanned the previously identified QTL regions in Lacaune, Churra and Sarda (Additional file 7: Figure S3).

### Annotation of SNP markers and QTL candidate regions

A relatively small number of genes (53 protein-coding and 26 microRNAs) were identified in the candidate regions for mastitis resistance (Additional file 8: Table S5). All significant SNP markers, with two exceptions, were located in intergenic or intronic regions. The exceptions were two SNPs associated significantly with TVC (oar3_OAR2_209,240,636) and SCC (Oar3_Oar2_208,650,955) in late lactation, which correspond to a missense variant in the exonic region of an enzyme of the citric acid cycle (isocitrate dehydrogenase 1, *IDH1*) and to a non-coding lincRNA variant, respectively. Annotations of all significant SNP markers for CM, SCC, CMT and TVC, are presented in Additional file 9: Table S6.

### Pathway and functional clustering analysis

**Fig. 3:**
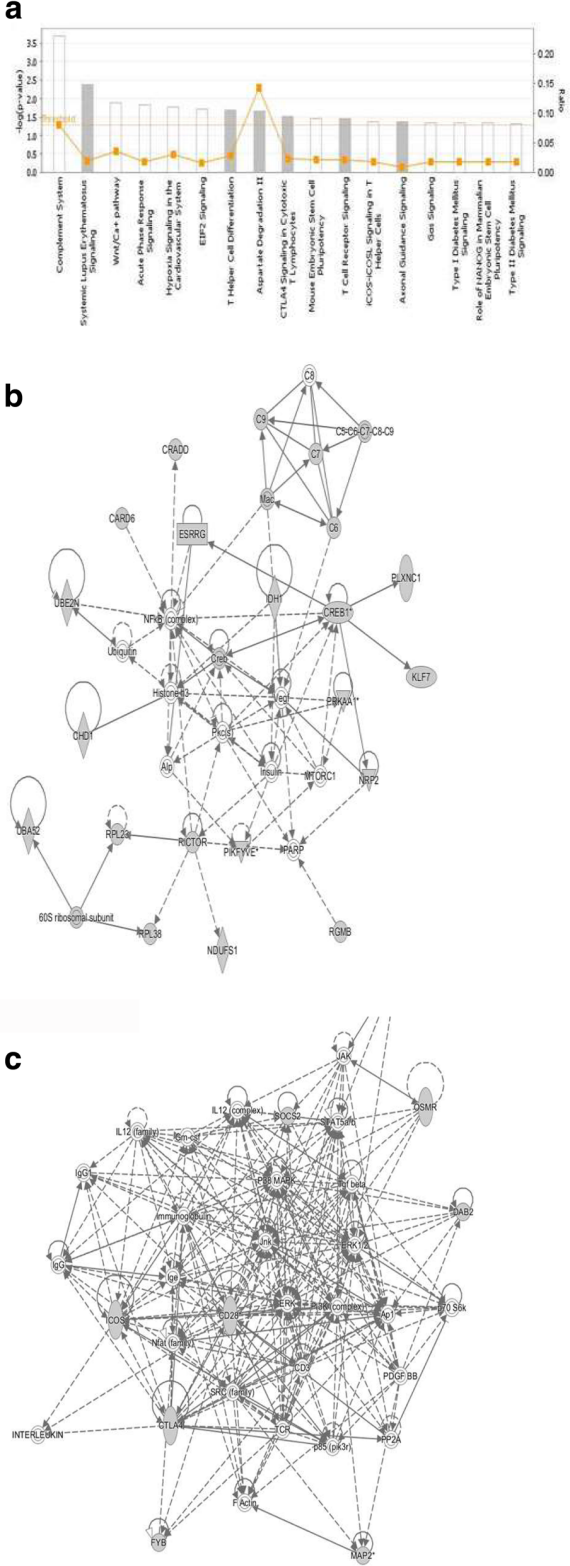
Pathway and network analysis using the IPA software. **a** The most highly represented canonical pathways derived from genes located within the candidate regions for mastitis resistance in Chios sheep. The solid yellow line represents the significance threshold. The line joining squares represents the ratio of the genes represented within each pathway to the total number of genes in the pathway. **b** Two networks, both related to immunological disease, that illustrate the molecular interactions between the products of candidate genes selected from QTL regions for mastitis resistance in Chios sheep. Arrows with solid lines represent direct interactions and arrows with broken lines represent indirect interactions. Genes with white labels are those added to the IPA analysis because of their interaction with the target gene products

Based upon the significant heritability estimates and the large amount of genetic variance accounted for by the identified SNPs, we reasoned that the corresponding QTL regions would contain genes contributing to a common pathway associated with mastitis resistance. We therefore identified the sets of annotated genes lying within QTL intervals and sought evidence of gene set enrichment. These genes were enriched for pathways involved in inflammatory and immune responses, both innate and adaptive (Fig. 3a). Moreover, two networks of molecular interactions related to immunological diseases were constructed using the list of genes in the candidate regions (Fig. 3b). We further extracted the gene ontology (GO) terms for each of the genes and performed functional annotation clustering analysis. These genes were organised into 18 clusters, each given an enrichment score (ES) (Additional file 10: Table S7). The first (ES = 2.33) and the third (ES = 1.53) clusters were related to the regulation of apoptosis, and to innate and adaptive immune responses involving the same immune genes identified with the pathway and network analyses (*C6, C8, C9, CD28, OSMR, SOCS2, CREB1, ICOS, CTLA4, NRP2, PRPKAA1, DAB2, LIFR, PTGER4, UBE2N, IDH1*).

### Gene expression analysis

Genes that contribute to mastitis resistance are likely to be expressed in milk somatic cells, in other immune cells, and/or in the mammary gland, and much of the genetic variation is likely to affect their transcriptional regulation. In humans, more than 80% of genes that are induced in stimulated monocytes have shown evidence of heritable variation in their level of expression (eQTL) [22]. To assess the expression profiles of genes located in the candidate regions for mastitis resistance, we obtained data from an ovine transcriptional atlas currently under development at the Roslin Institute, which is based upon the large-scale mRNA sequencing of multiple tissues and cell lines [23]; this development builds upon similar initiatives in other species [24, 25]. We also used data from an RNA-seq characterisation of the milk transcriptome of two Spanish dairy sheep breeds, Churra and Assaf, where milk somatic cells had been sampled at 10, 50, 120 and 150 days after lambing [26, 27]. A previous study identified genes involved in response to bacterial lipopolysaccharide (LPS), the major component of the outer membrane of Gram-negative bacteria, in a mouse mammary gland model of mastitis [28]. A similar model of mastitis has also been implemented in dairy cattle [29–31]. We reasoned that genes involved in mastitis susceptibility would likely be constitutively expressed in the mammary gland and immune tissues and/or differentially expressed in sheep bone marrow-derived macrophages (BMDMs) in response to LPS treatment. Both sources have been extensively analysed in the sheep atlas. In addition, we reasoned that these genes would be differentially expressed in sheep with high, intermediate and low SCC and/or their expression levels would correlate with SCC [32]. The sheep used in the milk transcriptomic study had also been phenotyped for SCC and milk yield [26, 27].

**Fig. 4:**
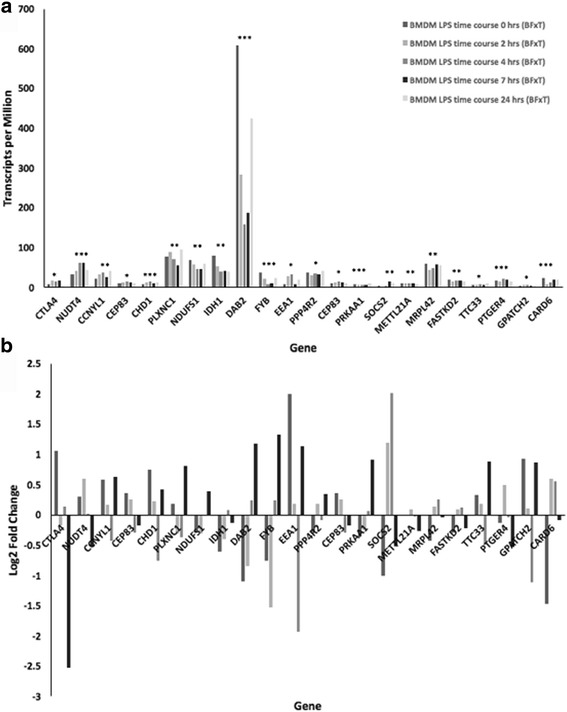
Differential expression of genes located in the candidate regions for mastitis resistance post treatment with LPS. Each column represents gene expression level in sheep bone marrow-derived macrophages 0, 2, 4, 7 and 24 h post treatment with LPS, respectively. Expression level is shown as (**a**) average TPM (transcripts per million) at the 5 different time points, and (**b**) as log_2_ fold change in expression at 2, 4, 7 and 24 h relative to expression at 0 h. Significance thresholds indicated as: *P* < 0.05*, *P* < 0.01** and *P* < 0.001***

Among all the genes located in the candidate regions for mastitis resistance identified in the present study, several (*DAB2, IDH1, NDUFS1, MRPL42, FYB, SOCS2, OSMR, RGMB, PPP4R2, EGFLAM* and *NUDT4)* were highly expressed in both mammary gland and various immune cell lines as shown in Additional file 11: Figure S4. These genes, along with others (*CTLA4, CCNYL1, CEP83, CHD1, TTC33, METTL21A, PLXNC1, CARD6, FASTKD2, PRKAA1* and *PTGER4*) were also inducible by LPS in sheep BMDMs (Fig. 4a and b).

**Fig. 5:**
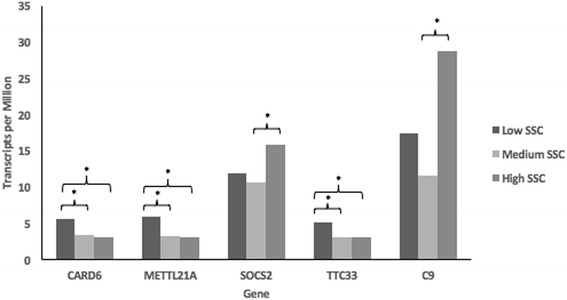
Differential expression of genes located in the candidate regions for mastitis resistance across individuals with low, medium and high milk somatic cell count. Expression in milk somatic cells is shown as average TPM (transcripts per million) across all experimental replicates. Significance values were estimated using Tukey’s HSD post hoc test (*P* < 0.05*)

Moreover, the expression of *CCNYL1, IDH1, SOCS2, MRPL42, C9, NDUFS1* and *NUDT4* was relatively higher than other genes in the milk transcriptome (Additional file 12: Figure S5). Some of the LPS-inducible genes (*SOCS2, CARD6, C9, METTL21A* and *TTC33*) were differentially expressed in the milk somatic cells of sheep with divergent levels of SCC (high, intermediate, low; Fig. 5). Furthermore, the level of expression of *OSMR, METTL21A, CEP83, CHD1, PLXNC1, GPATCH2, ICOS, PTGER4, CARD6* and *LIFR* were significantly associated with SCC, but not with milk yield levels, in the Churra and Assaf sheep.

### Transcription factor binding site (TFBS) analysis

Selective breeding in dairy sheep has led to the development of selective sweeps associated with milk production traits that are common to different breeds [33]. The apparent commonality of QTL regions affecting mastitis amongst disparate dairy breeds suggest that they may also share specific variants that confer this phenotype. There are currently insufficient whole genome sequences available to identify such variants on a population-wide basis. Nevertheless, we examined the available genomes of six meat sheep (Scottish Blackface x Texel; BFxT), the cross used to create the transcriptomic atlas, and six dairy sheep sequenced as part of the Sheep HapMap project [15] (two of each Churra, Sakiz and Lacaune breed). There is no publicly available Chios genome, but the genetically similar East Aegean Sakiz breed [20] was considered as a proxy in the present study. To focus on likely functional elements, we identified candidate TBFS within 1 kb of the annotated transcription start site [34]. MATCH analysis [35] revealed similar TFBS profiles between dairy and meat sheep in the majority (62%) of the genes located in the candidate regions for mastitis resistance. For the remaining genes, distinct TFBS profiles may be attributed to differences among individual animals rather than breed type (Additional file 13: Table S8). Most of these latter genes (*CHD1, PRKAA1, TTC33, CARD6, DAB2, FYB, OSMR, PPP4R2, C6, C9, PTGER4* and *LIFR*) were also differentially expressed in BMDMs after LPS treatment and/or in sheep with differing SCC. In general, no systematic differences were observed in TFBS profiles between dairy and meat sheep except for the following: (1) Mat1-Mc and AP-1 binding sites upstream of the *OSMR* and *PDZRN3* genes, respectively, only in dairy sheep; (2) an HNF-1 binding site upstream of *DAB2*, only in meat sheep; and (3) HSF and c-Rel sites upstream of *C9*, only in meat and dairy sheep, respectively.

### Selection of candidate genes

Based on all above results, a total of 14 genes were selected as the most promising candidate genes for mastitis resistance (Table 4) in the present study. Genes were selected using a combination of their known biological function, involvement in immune response pathways and networks, differential expression or enrichment in tissues relevant to mastitis, differences in TFBS, association of their expression levels with SCC and/or any previously known association with mastitis resistance in either dairy sheep or dairy cattle.

**Table 4:** Selected candidate genes for mastitis resistance

Gene Symbol	Gene name	Genomic location	Functions
*CTLA4*	Cytotoxic T-lymphocyte-associated protein 4	2: 204,777,523–204,784,522	Inhibitory receptor acting as a major negative regulator of T-cell responses [66].
*ICOS*	Inducible T-cell co-stimulator	2: 204,851,429–204,873,693	Enhances all basic T-cell responses to a foreign antigen, namely proliferation, lymphokine secretion, the upregulation of molecules that mediate cell-cell interactions, and antibody secretion by B-cells [67].
*IDH1*	Isocitrate dehydrogenase 1 (NADP+), Soluble	2:209,236,699–209,259,307	Isocitrate dehydrogenases catalyse the oxidative decarboxylation of isocitrate to 2-oxoglutarate; IDH1 also indirectly participates in mitigating oxidative damage and in the regulation of glucose-induced insulin secretion [68].
*SOCS2*	Suppressor of cytokine signalling 2	3:129,720,516–129,722,508	A member of the suppressor of cytokine signalling (SOCS) family. SOCS family members are cytokine-inducible negative regulators of cytokine receptor signalling via the Janus kinase/signal transducer and activation of transcription pathway (the JAK/STAT pathway) [69]. The protein encoded by this gene interacts with the cytoplasmic domain of insulin-like growth factor-1 receptor (IGF1R) and is thought to be involved in the regulation of IGF1R mediated cell signalling [70].
*PLXNC1*	Plexin C1	3:130,271,380–130,424,374	This gene encodes a member of the plexin family. Plexins are transmembrane receptors for semaphorins, a large family of proteins that regulate axon guidance, cell motility and migration, and the immune response [71].
*CHD1*	Chromodomain-helicase-DNA-binding protein 1	5:95,366,866–95,559,633	This gene encodes a protein that has been implicated in the regulation of transcription [72].
*C6*	Complement component 6	16:33,267,815–33,338,265	Constituents of the membrane attack complex, which plays a key role in the innate and adaptive immune response by forming pores in the plasma membrane of target cells [73].
*C7*	Complement component 7	16:33,481,056–33,542,035	
*C9*	Complement component 9	16:35,014,734–35,075,088	
*PTGER4*	Prostaglandin E receptor 4	16:33,690,480–33,707,155	This receptor can activate T-cell factor signalling, mediate PGE2 induced expression of early growth response 1 (EGR1), regulate the level and stability of cyclooxygenase-2 mRNA, and lead to the phosphorylation of glycogen synthase kinase-3 [74].
*DAB2*	Dab, mitogen-responsive phosphoprotein, homolog 2	16:34,976,596–34,998,498	Adapter protein that functions as a clathrin-associated sorting protein, required for the clathrin-mediated endocytosis of selected cargo proteins. Mediates the activation of transforming growth factor beta (TFG-beta)-stimulated c-Jun N-terminal kinases. May inhibit the canonical Wnt/beta-catenin signalling pathway. Plays a role in the colony stimulating factor 1 (CSF-1) signal transduction pathway. May also act as a tumour suppressor [75–77].
*FYB*	FYN-binding protein	16:35,170,143–35,269,324	The protein encoded by this gene is an adapter for the FYN protein and lymphocyte cytosolic protein 2 signalling cascades in T-cells. The encoded protein is involved in platelet activation and controls the expression of interleukin-2; it may also play a role in linking T-cell signalling to remodelling of the actin cytoskeleton [78].
*OSMR*	Oncostatin M receptor	16:35,426,282–35,474,718	Oncostatin M is a secreted cytokine involved in homeostasis and in diseases involving chronic inflammation. It is a member of the gp130 family of cytokines that have pleiotropic functions in differentiation and cell proliferation, as well as being involved in hematopoietic, immunologic, and inflammatory networks [79].
*CARD6*	Caspase Recruitment Domain	16:33,567,396–33,580,406	*CARD6* encodes a microtubule-associated protein that has been shown to interact with receptor-interacting protein kinases and to positively modulate signal transduction pathways converging on activation of the inducible transcription factor nuclear factor kB (NF-kB). It has a role in facilitating apoptosis [80, 81].

## Discussion

The present study investigated the genomic architecture of mastitis resistance in Chios dairy sheep and assessed the utility of relevant genomic data in genetic improvement. Five QTLs located on chromosomes 2, 3, 5, 16 and 19 that had been previously identified in three unrelated dairy sheep populations in France, Spain and Italy were found to be segregating in an independent Greek sheep breed, suggesting that genetic variation may persist in diverse populations and that joint genomic selection programmes for enhanced mastitis resistance across breeds are, in principle, feasible. The high proportion of genetic variance explained by the five common QTL regions renders this mastitis-specific 960SNP-array a useful tool in dairy sheep breeding and genetic improvement.

The significance of the QTL regions reported in the present study is supported by results of a previous selection mapping study that compared dairy with meat sheep breeds to identify genomic regions under selection [33]. In the latter study, multiple pairs of dairy versus meat sheep breeds were investigated. For two specific pairs a selection signal was identified in the QTL region for mastitis resistance on chromosome 16 reported in the present study; for another pair, a selection signal was also identified close to our QTL regions on chromosomes 2, 3 and 19. The consistency of these selective sweep signals with the QTLs verified in the present study attest to the significance and suitability of the genomic regions included in the custom-made 960SNP array for dairy sheep populations.

Substantial genetic variance was detected in all mastitis traits of our study. Nevertheless, our findings corroborate the notion that mastitis resistance is under mostly polygenic genetic control [7, 16, 36], since 60–70% of the genetic variance remained unexplained by the identified QTLs. Previous genetic studies on other breeds, reviewed by Bishop et al. [2], reported similar heritabilities ranging from 0.10 to 0.20 for SCC. Significant positive phenotypic and genetic correlations were detected between the CM occurrence and the three mastitis indicator traits (SCC, CMT and TVC) implying some common underlying mechanisms of resistance between acute clinical episodes and persistent intra-mammary inflammations. Similar correlations between CM and SCC have been reported in the Lacaune dairy sheep [32, 37] and cattle [38]. These results confirm the utility of the indirect mastitis measures as predictors of CM in genetic improvement programmes as well as on-farm management practices. Furthermore, the three mastitis indicator traits in the present study appeared to be under generally similar genetic mechanisms of control. Considering that the average incidence of clinical mastitis in a well-managed flock is about 2–3% and the incidence of subclinical mastitis is much higher, the use of indicator traits associated with the latter would facilitate breeding programmes achieving improvements in both subclinical and clinical mastitis resistance. CMT and TVC were studied for the first time as phenotypes for breeding purposes. Implementation of CMT would constitute a useful tool to phenotype mastitis resistance on the farm without requiring any special equipment.

In Lacaune sheep, a previous transcriptomic study of SCC associated 7 genes – cytotoxic T lymphocyte-associated protein 4 (*CTLA4*), suppressor of cytokine signalling 2 (S*OCS2*), oncostatin M receptor (*OSMR*), FYN oncogene related to SRC (*FYN*), complement factor B (*CFB*) and isocitrate dehydrogenase 2 (NADP+), soluble (*IDH2*) – with mastitis susceptibility [32]. In the present study, several of these genes (*CTLA4*, *SOCS2*, *OSMR*), along with some from the same family (*FYB*, *C9*, *IDH1*), were also found to be LPS-inducible in BFxT sheep BMDMs and/or differentially expressed in the milk somatic cells of Churra and Assaf, indicating they are likely to be involved in protective immunity across sheep populations. The SNP marker located within the *IDH1* gene corresponds to a missense mutation but further work is needed to verify if this is the actual causative mutation. For the *OSMR* and *C9* genes, differences in the TFBS in the 1 kb upstream regions were also identified among dairy and meat sheep. The proto-oncogene c-Rel – whose binding site was identified in the sequence upstream of *C9* in dairy sheep – is a member of the nuclear factor kappa b (NFkb) family of transcription factors, whose activity has been previously related with mastitis resistance in dairy cattle [39]. However, further studies are needed to confirm if these promoter variants are functionally important. Moreover, most of the candidate genes for mastitis resistance identified in the present study were previously reported to control mastitis resistance in dairy cattle, including *CTLA4* [40], *IDH1*, *OSMR*, *C6, C7* and *C9*, mitogen-responsive phosphoprotein, homolog 2 (*DAB2*), caspase recruitment domain family, member 6 *(CARD6*) [41, 42], and prostaglandin E receptor 4 (*PTGER4*) [43, 44]. This suggests that dairy sheep and cattle may partially share common underlying mechanisms and genes critical to a successful host response to mastitis infection.

Based upon their transcriptional profiles and association with SCC, novel functional candidates for mastitis resistance were identified in the present study. These include inducible T-cell co-stimulator (*ICOS*), leukaemia inhibitory factor receptor A (*LIFR*), plexin C1 (*PLXNC1*) and chromodomain-helicase-DNA-binding protein 1 (*CHD1*). The proteins encoded by these genes have been previously associated with multiple diseases in humans [45–49] and other species [50]. The clear enrichment of LPS-inducible genes in the QTL regions also offers the possibility of using this assay to identify both *cis*- and *trans*-acting eQTLs that are relevant to mastitis, similar to a previous study of human monocytes that highlighted loci associated with inflammatory disease susceptibility [22]. The fact that the set of LPS-inducible genes overlaps with genes whose level of expression was correlated with SCC attests to the utility of the assay as a model for mastitis in sheep. However, further studies are needed to confirm the present results and identify the actual causative genes and mutations.

## Conclusion

Both innate and adaptive immune responses, along with the induction of specific metabolic genes, are likely to be involved in the genomic architecture of sheep resistance to mastitis. All the mastitis traits analysed in the present study exhibited heritable variation, suggesting that selection and management programmes aiming at enhancing mastitis resistance are feasible. Genetic selection against mastitis may be achieved using primarily indirect (indicator) traits such as SCC, CMT and TVC but a combination of both clinical mastitis and indicator traits would be preferable. A possible implementation scenario might entail marker-assisted selection based on validated selectable markers and the candidate genes identified in the present study. Another option would be the implementation of genomic prediction and selection schemes across different dairy sheep breeds sharing common QTLs for mastitis resistance, leading to enhanced reference population size and greater accuracy.

## Methods

### Ethics statement

The study was approved by the Ethics and Research Committee of the Faculty of Veterinary Medicine, Aristotle University of Thessaloniki, Greece. Permits for access and use of the commercial farms were granted by the individual farm owners, who were members of the Chios Sheep Breeders’ Cooperative “Macedonia”. During sampling, animals were handled by qualified veterinarians. Permission to qualified veterinarians to perform milk and blood sampling was granted by the National (Greek) Legislature for the Veterinary Profession, No. 344/29–12-2000. For the ovine transcriptional atlas study approval was obtained from The Roslin Institute’s and the University of Edinburgh’s Protocols and Ethics Committees. All animal work was carried out under the regulations of the Animals (Scientific Procedures) Act 1986.

### Principal component analysis

A total of 23 Chios, 103 Lacaune, 120 Churra and 20 Sarda dairy sheep were analysed within the sheep HapMap project [15]. Genotyping and SNP quality control are detailed in Kijas et al. [20]. PCA was performed using PLINK v1.9 [51], which created an identity-by-state matrix for assessing the genetic differences among the four populations, and allowed the average genetic distance between Chios and each of the other three breeds to be estimated.

### Animals, sampling and phenotyping

A total of 609 purebred Chios dairy ewes in the first or second lactation, raised in four commercial farms in Greece, were used. Presence or absence (0/1) of CM was recorded by an experienced veterinarian in monthly visits during the first five months of lactation. On the day of visit, two 50 ml milk samples were collected in the milking parlour under aseptic conditions for the measurement of three traits indirectly related with mastitis: CMT, SCC and TVC. CMT was scored on a scale from 0 to 4 [52], with high values indicating the presence of elevated SCC and, potentially, pathogens in milk; this test was performed with a commercial kit according to manufacturer’s instructions (Bovi-vet, Kruuse, Germany). SCC was measured with Fossomatic 360 (Foss Electric, Hillerød, Denmark) and expressed as the number of cells/ml of milk. TVC was measured with Bactoscan FC 50 (Foss Electric, Hillerød, Denmark) and expressed as the number of viable bacteria/ml of milk. In total, 2436 individual animal records were collected. Peripheral blood samples were collected from each ewe in 9 ml K_2_EDTA Vacutainer blood collection tubes (BD diagnostics) by jugular venepuncture for genomic DNA extraction.

A total of 638 samples with a CMT score greater than or equal to 2 and/or at least 500,000 somatic cells/ml milk (cut-off values selected on the basis of bibliographic evidence [53]), were further tested by selective culturing on MacConkey agar for Gram-negative bacteria, and on Mannitol Salt agar and Blood agar followed by Gram staining for Gram positive bacteria. The plates were incubated at approximately 37 °C for 24 h. The next day, the plates were examined for bacterial growth.

### Genetic parameter estimation

The distributions of SCC and TVC records were skewed; therefore, records were naturally log-transformed in order to achieve normality of the respective distributions. Monthly animal records were analysed with the following mixed model to derive variance components and genetic parameters for the overall lactation; each trait was analysed separately:

1 $$ {Y}_{ijkmno}=\mu +{F}_i+{YS}_j+{a}_1\times age+{L}_k+\sum_{n= 1}^2{b}_n{P}_n{W}_m+{g}_o+{pe}_o+{e}_{ijkmno} $$

Where: Y = record of ewe *o* in week of lactation (i.e. week post-lambing) *m*

*μ* = overall mean

*F* = fixed effect of flock *i*

*YS* = fixed effect of year-season of lambing *j*

*α*_1_ = linear regression on age at lambing (*age*)

*L* = fixed effect of lactation number *k*

*W* = fixed effect of week of lactation *m*

*b*_*n*_ = fixed regression coefficient on week of lactation *m* (order *n* = 2)

*P*_*n*_ = orthogonal polynomial of week of lactation *m* (order *n* = 2)

*g* = random additive genetic effect of ewe *o*, including pedigree genetic relationships among animals

*pe* = random permanent environment effect of ewe *o*

*e* = random residual effect

A Logit function was fitted to the CM analysis to account for the binary nature (0/1) of the trait. For all traits, the fixed regression on week of lactation yielded a smoothed curve illustrating the trait progression throughout lactation. Heritability and repeatability estimates were derived from the variance components of the random effects for each trait. Residuals from these analyses were kept to be used as dependent variables in the ensuing genomic association studies aiming to assess the effect of SNPs on the respective traits expressed across the entire lactation. Subsequently, bivariate analyses were conducted with the above model to estimate genetic and phenotypic correlations among traits.

In separate analyses of SCC, CMT and TVC, second-order polynomial functions of week of lactation were fitted to the additive genetic and permanent environment effects of model (1), resulting in separate variance component and genetic parameter estimates by week of lactation. The latter were then combined to derive average heritability and repeatability estimates for early (weeks of lactation 1–7), mid (weeks 8–17) and late (weeks 18–23) lactation. The corresponding residuals were also kept as input variables to the genomic association studies aiming to assess the impact of SNPs on traits expressed in different stages of lactation. These analyses were not possible with the Logit function fitted to the analysis of binary CM. Therefore, a linear model was fitted to this trait in order to derive residuals by lactation stage for the genomic association analysis. All mixed model analyses were conducted with ASReml v4.0 [54].

### DNA extraction

Genomic DNA for all samples was extracted from blood buffy coat using a modified protocol (Modified Blood) as described by Psifidi et al. [55].

### Genomic association analysis

All animals were genotyped with a customized 960-SNP DNA array containing SNPs located in seven previously identified QTL regions for mastitis resistance on chromosomes 2, 3, 5, 12, 16 and 19. Details of the SNPs comprising the DNA array are presented in Additional file 14: Table S9. SNP genotypes were subjected to quality control measures using the following thresholds: minor allele frequency < 0.05, call rate < 95% and Hardy-Weinberg equilibrium *P* < 10^−6^. After quality control, 710 SNP markers remained for further analysis. SNP locations were obtained from the Oar v3.1 assembly using the Ensembl genome browser (www.ensembl.org). To account for possible population stratification, a genomic relationship matrix was generated including all individual animals. This genomic relationship matrix was converted to a distance matrix that was used to carry out classical MDS analysis with the R package GenABEL [56]. Individual ewe phenotypes were adjusted for the same fixed effects included in model (1). Phenotypes pertained to the entire course of lactation as well as to different stages (early, mid, late) of lactation as explained above. In all cases, GEMMA v0.94.1 [57] was used to run the genomic association analyses of adjusted phenotypes based on a mixed model that included the genomic relationship matrix among individual ewes as a random effect. After Bonferroni correction for multiple testing, the *P* ≤ 0.05 significance threshold was set at *P* ≤ 7.04 × 10^−5^ and a suggestive significance threshold (accounting for one false positive per genome scan) was set at *P* ≤ 1.41 × 10^−3^.

LD among significant SNPs was calculated as an *r* ^*2*^ value using PLINK v1.9 [51] in order to evaluate the extent of LD and identify candidate regions potentially containing causal mutations for mastitis resistance. Furthermore, LD blocks in the regions where significant SNPs were found with GWAS were visualised using the software Haploview v4.2 [58]

### SNP locus effect confirmation

Individual markers found to be significant in the previous step were further examined with a mixed model that included the fixed effects of model (1), the fixed effect of the corresponding SNP locus genotype and the random effect of the animal. Additive (a) and dominance (d) effects, and the proportion of additive genetic variance (PV_A_) and total phenotypic variance (PV_P_) due to each SNP locus were calculated as follows:

$$ {\displaystyle \begin{array}{l}\mathrm{a}=\left(\mathrm{AA}-\mathrm{BB}\right)/2\hfill \\ {}\mathrm{d}=\mathrm{AB}-\left(\left(\mathrm{AA}+\mathrm{BB}\right)/2\right)\hfill \\ {}{\mathrm{P}\mathrm{V}}_{\mathrm{A}}=\left(2\mathrm{pq}\ {\left(\mathrm{a}+\mathrm{d}\ \left(\mathrm{q}-\mathrm{p}\right)\right)}^2\right)/\mathrm{VA}\hfill \\ {}{\mathrm{P}\mathrm{V}}_{\mathrm{P}}=\left(2\mathrm{pq}\ {\left(\mathrm{a}+\mathrm{d}\ \left(\mathrm{q}-\mathrm{p}\right)\right)}^2\right)/\mathrm{VP}\hfill \end{array}} $$

where AA, BB and AB were the predicted trait values of the respective genotypic classes, p and q were the allelic frequencies of A and B at the SNP locus, and VA and VP were the additive genetic and total phenotypic variance of the trait. The latter were estimated with model (1). All analyses were run with ASReml v4.0 [54].

### Annotation of the SNP markers and the QTL candidate regions

For sheep assembly Oar v3.1, the Ensembl Variant Effect Predictor (VEP) (http://www.ensembl.org/Tools/VEP) and BioMart data mining tools (http://www.ensembl.org/biomart/martview/) were used to map and annotate the significant SNP markers derived with the genomic association analysis on the reference genome, and to locate genes in the corresponding candidate regions for mastitis resistance.

### Pathway and functional annotation clustering analysis

Gene lists in the candidate regions for mastitis resistance were analysed using the Ingenuity Pathway Analysis (IPA) programme (www.ingenuity.com) in order to identify canonical pathways and gene networks constructed by the products of these genes. IPA constructs multiple possible upstream regulators, pathways and networks serving as hypotheses for the biological mechanism underlying the trait. This analysis uses data from a large-scale causal network derived from the Ingenuity Knowledge Base. IPA then infers the most suitable pathways and networks based on their statistical significance, thereby setting a threshold above which the pathways are significant.

Gene lists were also analysed against an *Ovis aries* background using the Database for Annotation, Visualization and Integrated Discovery (DAVID v6.7) [59]. For each gene, we determined its GO terms and performed functional annotation clustering analysis to detect possible enrichment. The ES calculated with DAVID is a modified Fisher’s exact test *P*-value, with increasing ES (>1) reflecting over-representation of that functional category.

### Gene expression analysis

Expression levels of each gene located within candidate regions for a mastitis trait were obtained from a transcriptomic atlas of 406 RNA-seq libraries, representing all major organ systems; the atlas was based on a BFxT sheep cross [23] and also included 83 libraries from a Texel sheep trio (ewe, ram and lamb) as described in Jiang et al. [60]. In the present study, we were specifically interested in mammary gland, immune-related tissues and cell lines. Therefore, we extracted data pertaining to the haemolymph nodes, mesenteric, popliteal, prescapular and submandibular lymph nodes, peripheral blood mononuclear cells, blood leukocytes, monocyte-derived macrophages, alveolar macrophages, tonsils, and mammary glands. The atlas also contained data from sheep BMDMs at different time points (0 h, 2 h, 4 h, 7 h and 24 h) after LPS treatment [23], which we used here as a model for mastitis, to identify candidate genes that are likely involved in the inflammatory response to bacterial infection. LPS stimulation mimics Gram-negative pathogens, which accounted for 12% of the mastitis incidence in the Chios sheep in the present study. LPS stimulation also provides a general model for toll-like receptor (TLR) signalling and inflammatory pathology which causes morbidity and production losses associated with mastitis. To supplement this dataset, we obtained expression levels from a previous transcriptomic study of the milk somatic cells of two Spanish dairy sheep breeds, Churra and Assaf [27]. The sheep used in this study had also been phenotyped for SCC and milk yield [26]; these records were made available to the present study.

To generate RNA for library preparation, BMDMs were differentiated for 7 days in recombinant human colony stimulating factor and treated with LPS (*Salmonella minnesota* Re 595 (L9764; Sigma-Aldrich)) at a concentration of 100 ng/ml. *Salmonella* is a TLR4 agonist; *Staphylococcus aureus*, which is the main cause of mastitis in dairy sheep, is known to activate both TLR2 and TLR4 [61] in cattle. BMDMs were harvested into Trizol (Thermofisher) at each different time point post LPS treatment in preparation for RNA extraction. For all samples in the transcriptomic atlas, RNA was extracted using Trizol (Thermofisher) followed by column purification and DNAse treatment using the RNeasy Mini Kit (Qiagen). The resultant RNA was checked for quality using the Agilent Tapestation 2200, and all samples were of high quality with RNA Integrity Numbers (RIN^e^) greater than 9. All RNA-Seq libraries from the sheep transcriptional atlas included in this study were TruSeq stranded mRNA libraries (125 bp paired-end). Libraries were prepared by Edinburgh Genomics (https://genomics.ed.ac.uk/) using the TruSeq mRNA Library Preparation kit (Illumina) and sequenced on the Illumina HiSeq v2500 at a depth of >25 million reads per sample.

Expression levels for all samples from both the transcriptomic atlas and the milk somatic cell transcriptome were estimated using Kallisto v0.42.4 [62]. Rather than aligning RNA-seq reads to a reference genome, reconstructing transcripts from these alignments and then quantifying expression as a function of the reads aligned (the conventional means of RNA-seq processing), Kallisto employs a ‘lightweight’ algorithm, which first builds an index of k-mers from a known transcriptome: the *Ovis aries* v3.1 cDNAs (ftp://ftp.ensembl.org/pub/release-83/fasta/ovis_aries/cdna/Ovis_aries.Oar_v3.1.cdna.all.fa.gz, obtained from Ensembl v84 [63]; *n* = 23,113 transcripts [22,823 protein-coding, 247 pseudogenes, 43 processed pseudogenes]). Expression level is then estimated directly (i.e., in an alignment-free manner) by quantifying exact matches between reads and k-mers. Expression is reported per transcript as the number of transcripts per million, and is summarised to the gene-level as described previously [64].

Differential expression analysis was run on the LPS time series data using the Kallisto output with the R/Bioconductor package ‘Sleuth’. Heatmaps were drawn using the heatmap.2 function from the R package gplots v3.0.1. Additional differential expression analyses were conducted between sheep with high, moderate and low levels of SCC, after adjusting for breed (Churra and Assaf). Relevant data for this study are described by Suarez-Vega et al. [22]. Least square mean pairwise comparisons between SCC levels were conducted. Tukey’s HSD post-hoc test adjustment was applied at a significance level of 0.05.

Furthermore, the effect of the expression level of each gene (located within a candidate region for mastitis resistance) on SCC and milk yield was assessed using the following linear model:

2 $$ Yij=\mu +{B}_i+{g}_j+ eij $$

where Y = record of ewe (SCC or milk yield across lactation), *μ* = overall mean, *B* = fixed effect of breed *i*, *g* = fixed effect of the mean expression of gene *j*, *e* = random residual effect.

The significance threshold in this analysis was set at 0.05. Since genes were located within 5 QTL regions, an FDR adjustment for multiple testing was applied and the significance threshold was subsequently re-set to *P* ≤ 0.0125. The analyses were conducted with ASReml v4.0 [36].

### Transcription factor binding site analysis

In order to identify whether differences in TFBS are associated with differences in expression level of the genes found in the candidate regions for mastitis resistance, we extracted and compared the corresponding 1 kb upstream sequence in all genes from both meat and dairy sheep. Specifically, we extracted the sequences from the six sheep used to create the BFxT transcriptomic atlas and six sheep sequenced as part of the Sheep HapMap Project [15] (two Churra, two Lacaune [one dairy and one meat] and two Sakiz dairy sheep; NCBI Sequencing Read Archive (SRA) accession numbers SRR501848, SRR501909, SRR501850, SRR501851, SRR501843 and SRR501878, respectively; BioProject PRJNA160933).

The six atlas sheep (BFxT) had been fully re-sequenced. Illumina Tru-Seq Nano 350 gel-free libraries (125 bp paired end) were prepared, for each sample, by Edinburgh Genomics (https://genomics.ed.ac.uk). The six libraries were run on one lane of the Illumina HiSeq2500 System to generate in total approximately 220 M + 220 M reads resulting in a 10-fold coverage per sample. Details of the re-sequencing methodology within the HapMap sheep project can be found in Kijas et al. [15]. In both cases, the 1 kb upstream regions for each gene were extracted using BEDTools v2.25 [65]. These were then analysed using the MATCH algorithm within the TRANSFAC^®^ 7.0 Suite, with default parameters, which predicts TFBS in DNA sequences using a library of positional weight matrices [35].

## Additional files

Additional file 1: Table S1. Location of identified QTL regions for mastitis resistance in Chios sheep (present study) as well as Lacaune, Churra and Sarda sheep from previous studies. (XLSX 13 kb)

Additional file 2: Figure S1. Principal component analysis results for Chios, Lacaune, Churra and Sarda sheep. Red dots represent Chios (present study), green dots Churra, blue dots Lacaune and orange dots Sarda sheep (previous studies). (DOCX 20 kb)

Additional file 3: Table S2. Descriptive statistics of milk somatic cell count (SCC), California mastitis test (CMT) and total viable bacterial count in milk (TVC) in Chios sheep. (DOCX 12 kb)

Additional file 4: Table S3. Genetic correlations among mastitis measures in different lactation stages in Chios sheep. (DOCX 12 kb)

Additional file 5: Figure S2. Q-Q plots displaying the genomic association results for the mastitis traits studied in Chios sheep. Q-Q plots for milk somatic cell count (SCC) in early (A), late (B) and overall (C) lactation; for California mastitis test (CMT) in early (D), mid (E), late (F) and overall (G) lactation; for total viable bacterial count in milk (TVC) in early (H), late (I) and overall (J) lactation; for clinical mastitis occurrence (CM) in early (K), mid (L), late (M) and overall (N) lactation; Observed *P*-values are plotted against the expected *P*-values for each trait. Q-Q plots are not presented for TVC and SCC in mid lactation since no significant results for these traits were identified. (DOCX 248 kb)

Additional file 6: Table S4. Linkage disequilibrium (LD) estimation (as *r* ^*2*^) for the significant SNP markers identified in the genomic association analyses of mastitis resistance in Chios sheep. (XLSX 13 kb)

Additional file 7: Figure S3. Patterns of linkage disequilibrium (LD) for SNP markers associated significantly with mastitis resistance in Chios sheep. LD patterns are shown for chromosomes 2 (A), 3 (B), 5 (C), 16 (D) and 19 (E). LD blocks are marked with triangles. The significant markers are illustrated with a blue arrow. The candidate regions for mastitis resistance identified in previous studies and in Chios sheep (present study) are marked with dashed lines. The strongest LD signals are in red and the weakest in white. (DOCX 1060 kb)

Additional file 8: Table S5. Genes located in the candidate genomic regions for mastitis resistance in Chios sheep. (XLSX 15 kb)

Additional file 9: Table S6. Annotation of SNPs identified to have a significant association with mastitis resistance traits. (XLSX 16 kb)

Additional file 10: Table S7. Functional annotation clustering analysis of the genes located in the candidate regions for mastitis resistance. (XLSX 16 kb)

Additional file 11: Figure S4. Expression level of genes, located in the mastitis resistance candidate regions, across both mammary glands and immune cell lines/tissues. Expression level is estimated as the mean TPM (transcripts per million) of all (5) experimental replicates and is represented here as a Z-score per cell line/tissue. Data is obtained from two transcriptomic atlases; one of Scottish Blackface x Texel (BFxT) sheep and one of Texel sheep. (PNG 67 kb)

Additional file 12: Figure S5. Expression level of genes, located in the mastitis resistance candidate regions, as extracted from the Churra/Assaf milk somatic cell transcriptome analysis. Expression level is estimated as the mean TPM (transcripts per million) of all (5) experimental replicates and is represented here as a Z-score per individual animal. (PNG 54 kb)

Additional file 13: Table S8. Transcription factor binding site analysis using the MATCH programme. (XLSX 41 kb)

Additional file 14: Table S9. List of SNP markers comprising the mastitis-specific custom-made array used in the genomic association analysis of Chios sheep. (XLSX 51 kb)

Additional file 15: Table S10. Expression levels of genes located in the candidate regions for mastitis resistance identified in Chios sheep. The gene expression levels presented here are across all the body tissues and cell lines, as extracted from the two (BFxT and Texel) sheep transcriptomic atlases. Details on the expression profile of each gene across the transcriptomic atlas are also provided. (XLSX 71 kb)

## Abbreviations

BFxT: Scottish Blackface x Texel
BMDMs: bone marrow derived macrophages
*C6, C7 and C9*: complement components 6, 7 and 9
*CARD6*: caspase recruitment domain family, member 6
*CFB*: complement factor B
*CHD1*: chromodomain-helicase-DNA-binding protein 1
CM: clinical mastitis occurrence
CMT: California mastitis test
*CTLA4*: cytotoxic T lymphocyte-associated protein 4
*DAB2*: mitogen-responsive phosphoprotein, homolog 2
DAVID: Database for Annotation, Visualization and Integrated Discovery
Dst: pairwise genetic distance
*E. coli*: *Escherichia coli*
eQTL: expression quantitative trait loci
ES: enrichment score
*FYN*: FYN oncogene related to SRC
GO: gene ontology
*ICOS*: inducible T-cell co-stimulator
*IDH1*: isocitrate dehydrogenase 1 (NADP+), soluble
*IDH2*: isocitrate dehydrogenase 2 (NADP+), soluble
IPA: Ingenuity Pathway Analysis
LD: linkage disequilibrium
*LIFR*: leukaemia inhibitory factor receptor A
LPS: lipopolysaccharide
MDS: multidimensional scaling analysis
*OSMR*: oncostatin M receptor
PCA: principal component analysis
*PLXNC1*: plexin C1
*PTGER4*: prostaglandin E receptor 4
QTL: quantitative trait locus
*S. aureus*: *Staphylococcus aureus*
SCC: milk somatic cell count
SNP: single nucleotide polymorphism
*SOCS2*: suppressor of cytokine signalling 2
SRA: NCBI Sequencing Read Archive
TFBS: Transcription factor binding site
TVC: total viable bacterial count in milk
VEP: Variant Effect Predictor
