# Supplementary figures and images for: The genomic architecture of mastitis resistance in dairy sheep
Source: BMC Genomics. 2017 Aug 16;18:624. doi: 10.1186/s12864-017-3982-1 (PMC5559839; doi:10.1186/s12864-017-3982-1)

Chios: **red**

Churra: **green**

Lacaune: **blue**

Sardinian: **orange**

PCA1 and PCA2 explained 14.4% and 9.9% of the variance


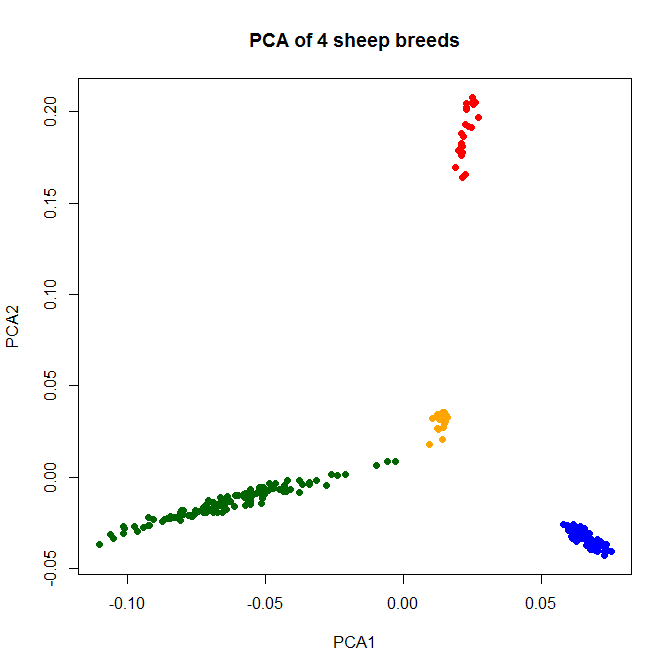

Supplement: Supplementary file 2 — Principal component analysis results for Chios, Lacaune, Churra and Sarda sheep. Red dots represent Chios (present study), green dots Churra, blue dots Lacaune and orange dots Sarda sheep (previous studies). (DOCX 20 kb) [file 12864_2017_3982_MOESM2_ESM.docx]

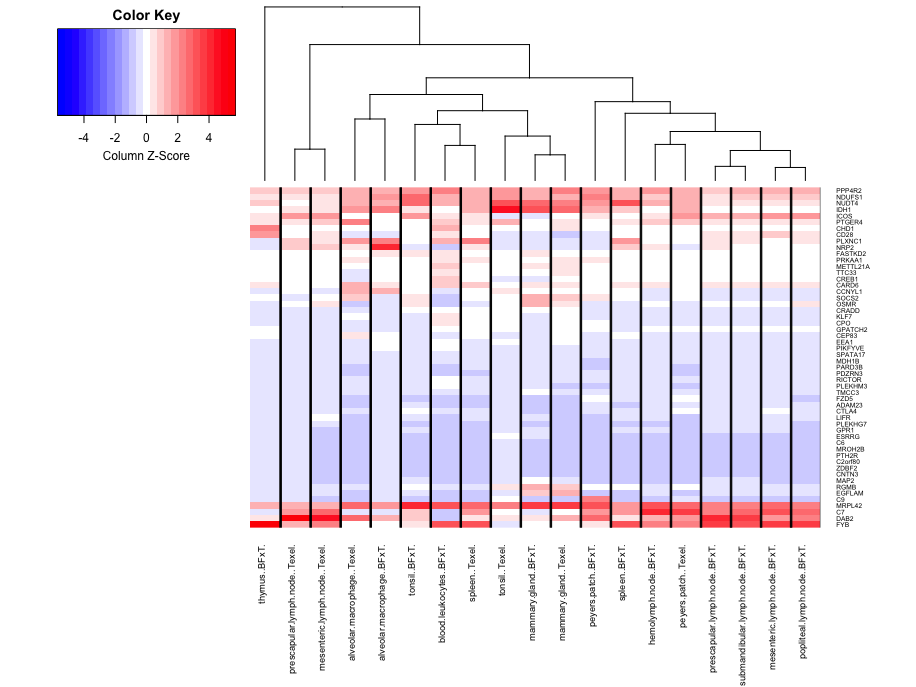

Supplement: Supplementary file 11 — Expression level of genes, located in the mastitis resistance candidate regions, across both mammary glands and immune cell lines/tissues. Expression level is estimated as the mean TPM (transcripts per million) of all (5) experimental replicates and is represented here as a Z-score per cell line/tissue. Data is obtained from two transcriptomic atlases; one of Scottish Blackface x Texel (BFxT) sheep and one of Texel sheep. (PNG 67 kb) [file 12864_2017_3982_MOESM11_ESM.png]

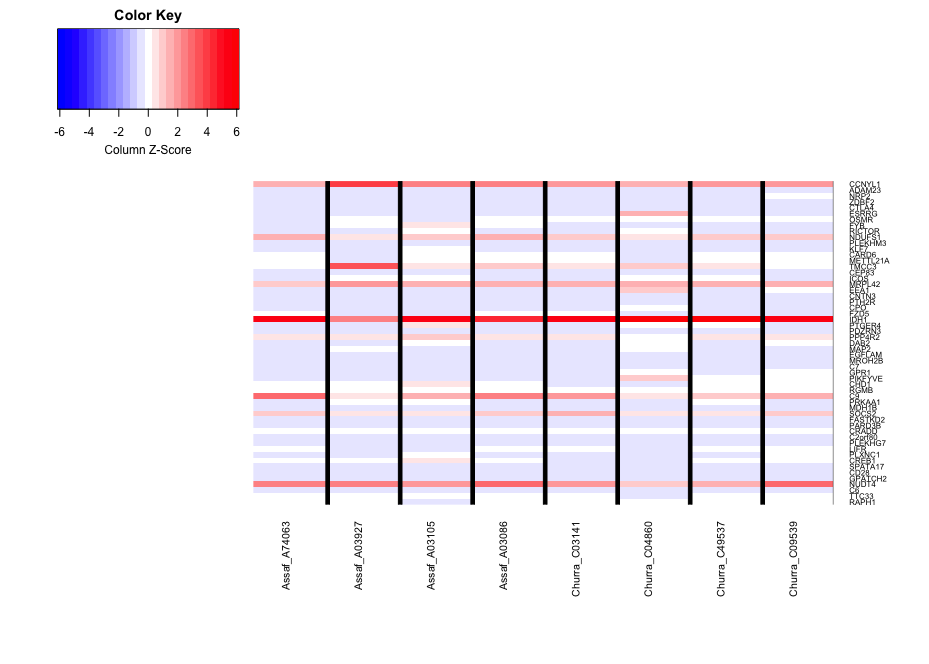

Supplement: Supplementary file 12 — Expression level of genes, located in the mastitis resistance candidate regions, as extracted from the Churra/Assaf milk somatic cell transcriptome analysis. Expression level is estimated as the mean TPM (transcripts per million) of all (5) experimental replicates and is represented here as a Z-score per individual animal. (PNG 54 kb) [file 12864_2017_3982_MOESM12_ESM.png]
