# Supplementary material for: The genomic architecture of mastitis resistance in dairy sheep
Source: BMC Genomics. 2017 Aug 16;18:624. doi: 10.1186/s12864-017-3982-1 (PMC5559839; doi:10.1186/s12864-017-3982-1)
Supplement: Supplementary file 3 — Descriptive statistics of milk somatic cell count (SCC), California mastitis test (CMT) and total viable bacterial count in milk (TVC) in Chios sheep. (DOCX 12 kb) [file 12864_2017_3982_MOESM3_ESM.docx]

|  | Mean | | Standard deviation | | Minimum value | | Maximum value | |
| --- | --- | --- | --- | --- | --- | --- | --- | --- |
| SCC (×10^3^ cells/ml) | 392 | 674 | | 16 | | 3,939 | |  |
| CMT (0-4 scale) | 0.84 | 1.38 | | 0 | | 4 | |  |
| TVC (×10^3^cfu/ml) | 19 | 51 | | 8 | | 771 | |  |
