# Supplementary material for: The genomic architecture of mastitis resistance in dairy sheep
Source: BMC Genomics. 2017 Aug 16;18:624. doi: 10.1186/s12864-017-3982-1 (PMC5559839; doi:10.1186/s12864-017-3982-1)
Supplement: Supplementary file 4 — Genetic correlations among mastitis measures in different lactation stages in Chios sheep. (DOCX 12 kb) [file 12864_2017_3982_MOESM4_ESM.docx]

| Trait | Lactation Stage | early | | mid | |
| --- | --- | --- | --- | --- | --- |
| SCC | mid | 0.81 | (0.03) |  |  |
|  | late | 0.49 | (0.07) | 0.88 | (0.02) |
| TVC | mid | 0.69 | (0.07) |  |  |
|  | late | 0.26 | (0.13) | 0.81 | (0.05) |
| CMT | mid | 0.84 | (0.02) |  |  |
|  | late | 0.56 | (0.06) | 0.89 | (0.02) |
| SCC: milk somatic cell count; CMT: California Mastitis Test; TVC: total viable bacterial count in milk | | | | | |
