# Supplementary material for: The genomic architecture of mastitis resistance in dairy sheep
Source: BMC Genomics. 2017 Aug 16;18:624. doi: 10.1186/s12864-017-3982-1 (PMC5559839; doi:10.1186/s12864-017-3982-1)

1. Q-Q plot for somatic cell count in milk (SCC) in early lactation


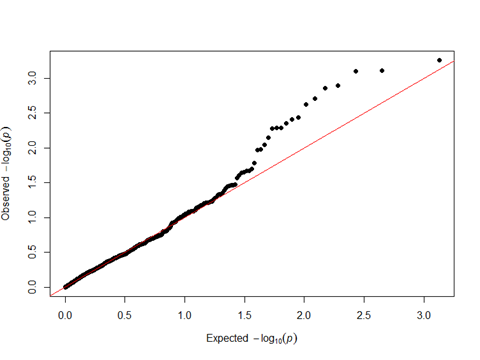


1. Q-Q plot for SCC in late lactation


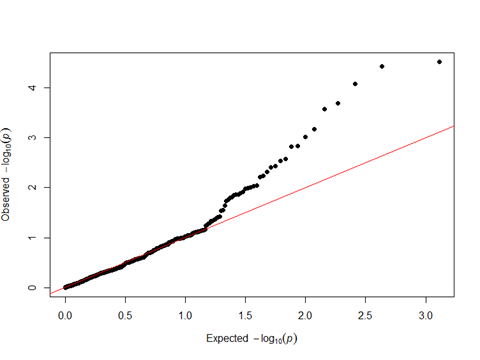


1. Q-Q plot for SCC in overall lactation


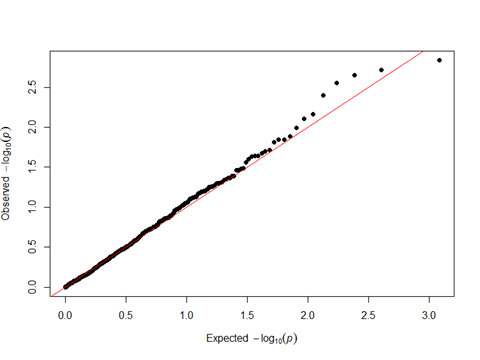


D) Q-Q plot for California mastitis test (CMT) in early lactation


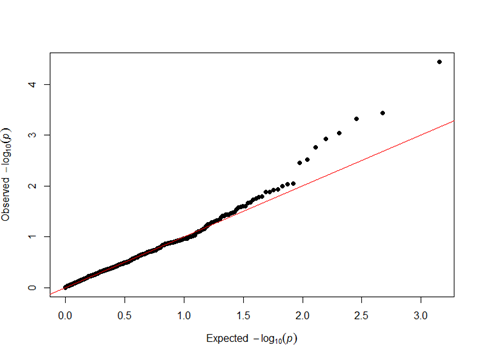


E) Q-Q plot for CMT in mid lactation


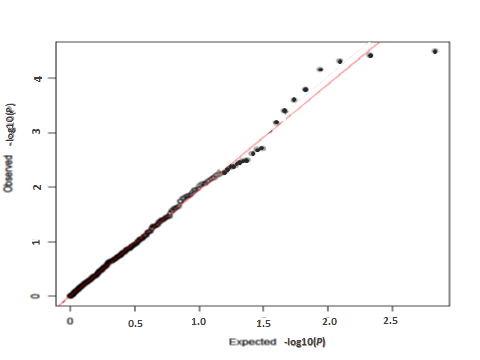


1. Q-Q plot for CMT in overall lactation
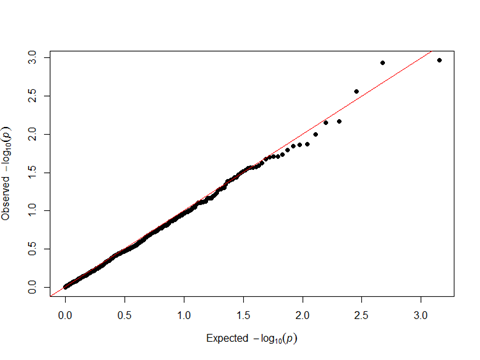

2. Q-Q plot for total viable bacterial count in milk (TVC) in early lactation


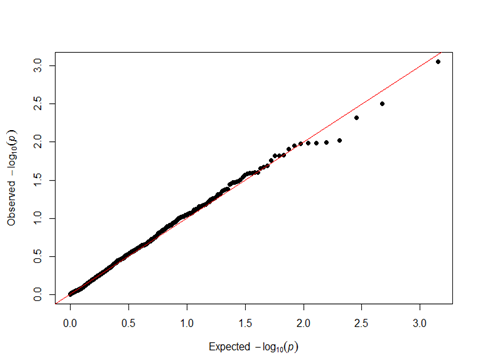


1. Q-Q plot for TVC in late lactation


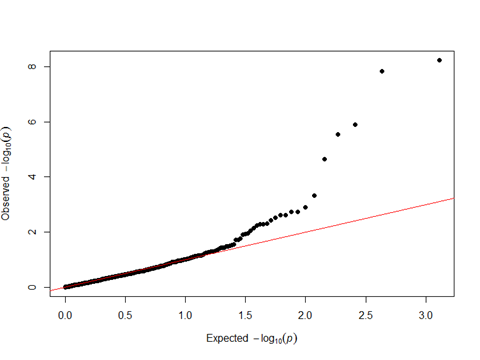


1. Q-Q plot for TVC in overall lactation


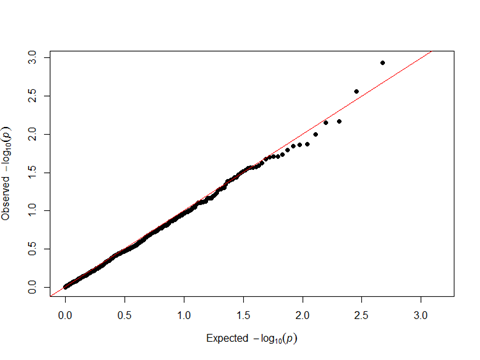


1. Q-Q plot for clinical mastitis occurrence (CM) in early lactation


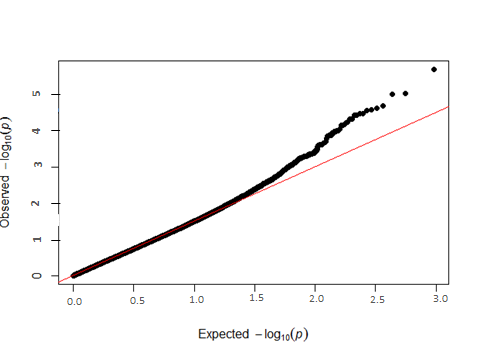


1. Q-Q plot for CM in mid lactation


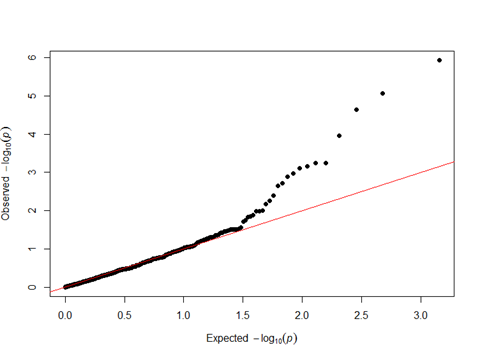


1. Q-Q plot for CM in late lactation


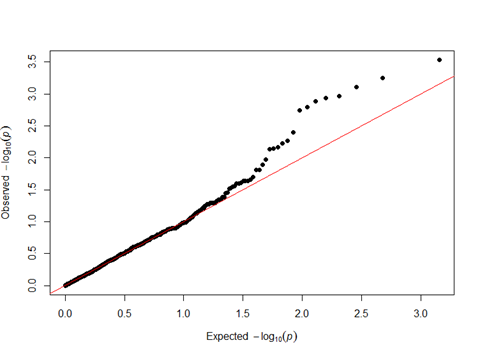


1. Q-Q plot for CM in overall lactation


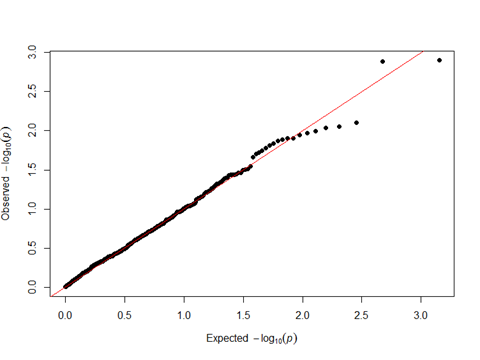


1. Q-Q plot for total viable bacterial count in milk (TVC) in early lactation


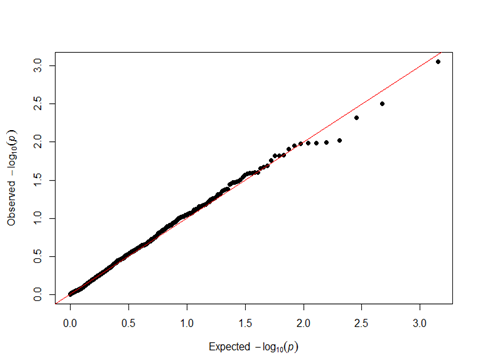


1. Q-Q plot for TVC in late lactation


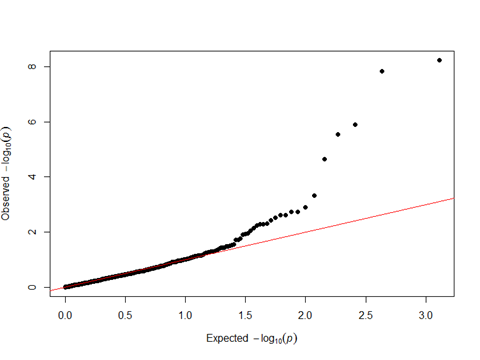


1. Q-Q plot for TVC in overall lactation


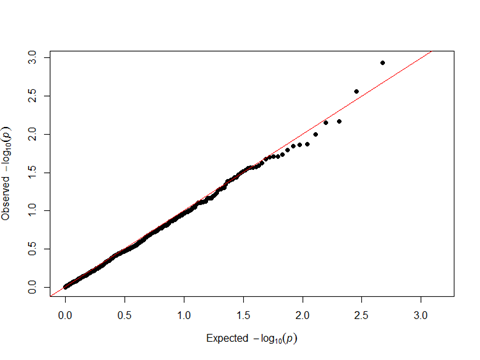

Supplement: Supplementary file 5 — Q-Q plots displaying the genomic association results for the mastitis traits studied in Chios sheep. Q-Q plots for milk somatic cell count (SCC) in early (A), late (B) and overall (C) lactation; for California mastitis test (CMT) in early (D), mid (E), late (F) and overall (G) lactation; for total viable bacterial count in milk (TVC) in early (H), late (I) and overall (J) lactation; for clinical mastitis occurrence (CM) in early (K), mid (L), late (M) and overall (N) lactation; Observed P-values are plotted against the expected P-values for each trait. Q-Q plots are not presented for TVC and SCC in mid lactation since no significant results for these traits were identified. (DOCX 248 kb) [file 12864_2017_3982_MOESM5_ESM.docx]
