# Supplementary material for: The genomic architecture of mastitis resistance in dairy sheep
Source: BMC Genomics. 2017 Aug 16;18:624. doi: 10.1186/s12864-017-3982-1 (PMC5559839; doi:10.1186/s12864-017-3982-1)

1. Linkage disequilibrium pattern on chromosome 2


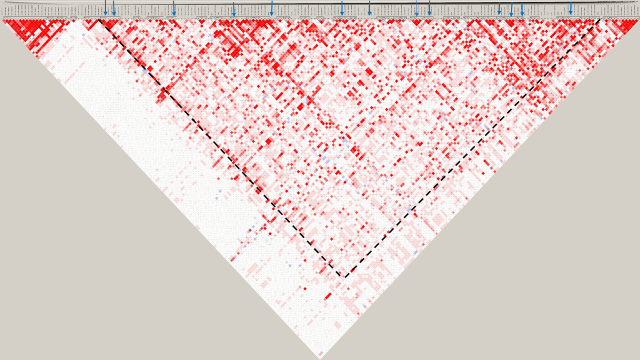


1. Linkage disequilibrium pattern on chromosome 3


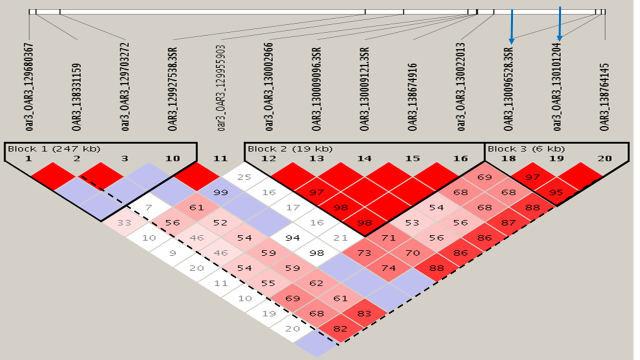


1. Linkage disequilibrium pattern on chromosome 5


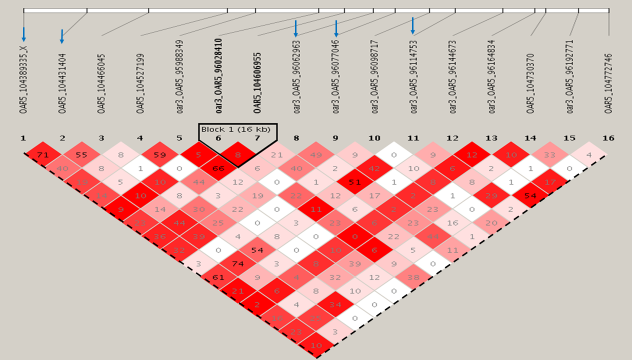


1. Linkage disequilibrium pattern on chromosome 16


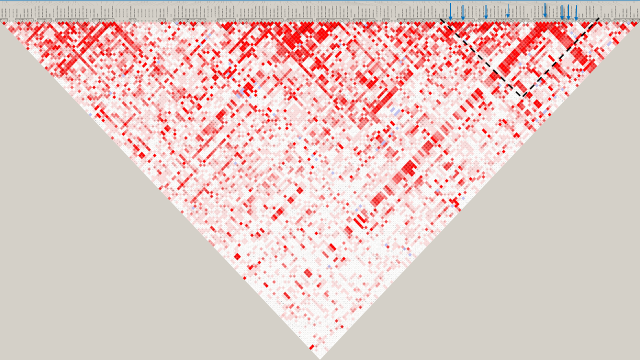


1. Linkage disequilibrium pattern on chromosome 19


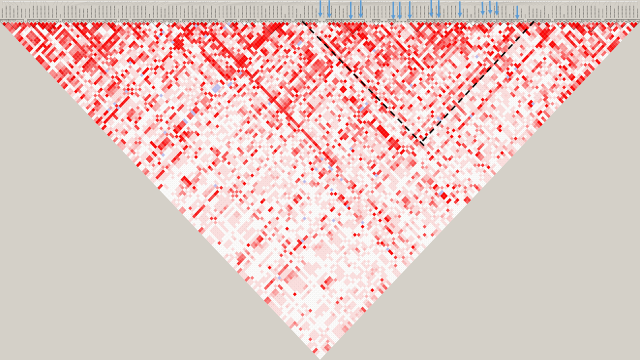

Supplement: Supplementary file 7 — Patterns of linkage disequilibrium (LD) for SNP markers associated significantly with mastitis resistance in Chios sheep. LD patterns are shown for chromosomes 2 (A), 3 (B), 5 (C), 16 (D) and 19 (E). LD blocks are marked with triangles. The significant markers are illustrated with a blue arrow. The candidate regions for mastitis resistance identified in previous studies and in Chios sheep (present study) are marked with dashed lines. The strongest LD signals are in red and the weakest in white. (DOCX 1060 kb) [file 12864_2017_3982_MOESM7_ESM.docx]
